# Data on the relationships between financing strategies, entrepreneurial competencies and business growth of technology-based SMEs in Nigeria

**DOI:** 10.1016/j.dib.2018.03.136

**Published:** 2018-04-03

**Authors:** Ayodotun Stephen Ibidunni, Oladele Joseph Kehinde, Oyebisi Mary Ibidunni, Maxwell Ayodele Olokundun, Falola Hezekiah Olubusayo, Odunayo Paul Salau, Taiye Tairat Borishade, Peter Fred

**Affiliations:** a Department of Business Management, Covenant University, Ota, Ogun State, Nigeria; b Department of Accounting, Bells University of Technology, Ota, Ogun State, Nigeria

**Keywords:** Financing strategies, Entrepreneurial competencies, Technology-based SMEs, SMEs business growth, SME performance

## Abstract

The article presents data on the relationship between financing strategies, entrepreneurial competencies and business growth of technology-based SMEs in Nigeria. Copies of structured questionnaire were administered to 233 SME owners and financial managers. Using descriptive and standard multiple regression statistical analysis, the data revealed that venture capital and business donations significantly influences profit growth of technology-based SMEs. Moreover, the data revealed that technology-`based firms can enhance their access to financing through capacity building in entrepreneurial competencies, such as acquiring the right skills and attitude.

**Specifications Table**

**Table:** 

Subject area	*Strategic Management, Entrepreneurship*
More specific subject area	*Entrepreneurial Competencies, SMEs, Technology Management*
Type of data	*Table, figure*
How data was acquired	*Researcher made questionnaire analysis*
Data format	*Raw, analyzed, descriptive and statistical data*
Experimental factors	– *Samples consist of Owner and Financial managers of technology-based SMEs in Lagos State, Nigeria.* – *In this paper, the relationship between financing strategies, entrepreneurial competencies and growth of technology-based SMEs was examined.*
Experimental features	*Understanding the hierarchical importance of financing strategies and the influence of entrepreneurial competencies is a critical factor for enhancing the growth of technology-based SMEs*
Data source location	*Owner and Financial managers of technology-based SMEs in Lagos State, Nigeria*
Data accessibility	*Data is included in this article*

**Value of the data**

• The data presented describe demographic attributes of owners and financial managers of technology-based SMEs, especially those in Lagos state. This could be used by other researchers.
• The data describe the hierarchical order of importance of financing towards enhancing the growth of technology-based SMEs in Nigeria. Consequently, the data could provide insights for other researchers.
• The data allows other researchers to extend the statistical analysis.

## 1 Data

Table 1 shows that 52.6% (121) of the respondents are male and 47.4% (109) of the respondents are female. 21.4% (47) of the respondents are single, 56.8% (125) of the respondents are married, while 20.6%(48) respondents are in other categories, such as divorced, widowed or separated. 29.8% (67) of the respondents are between the age of 21–30 years, 57.8% (130) of the respondents are between the age of 30–40 years, 8.9% (20) of the respondents are between the age of 41–50 years and 3.6% (8) of the respondents are 51 years and above. 33% (72) of the respondents have WAEC/O Level results as their highest educational qualification, 27.5%(60) of the respondents have OND/NCE as their highest educational qualification, 25.7% (56) of the respondents have B.SC/HND as their highest educational qualification, 12.8% (30) of the respondents have obtained a postgraduate degree.

**Table 1:** Demographic characteristics of respondents.

Parameter	Characteristics	Number (Percentage)
Gender	Male	121 (51.9)
	Female	109 (46.8)
Marital status	Single	47 (20.2)
	Married	125 (53.6)
	Others	48 (20.6)
Age bracket	21–30	67 (28.8)
	30–40	130 (55.8)
	41–50	20 (8.6)
	51 years and above	8 (3.4)
Educational qualification	WAEC/O’level	72 (30.9)
	NCE/HND	60 (25.8)
	HND/BSC	56 (24.0)
	Postgraduate	30 (12.8)

Table 2 contains data on the effect of financing strategies on technology-based SMEs’ business growth. It also reflects which financing strategy is perceived to have the greatest contribution to technology-based SMEs business growth. From the data, venture capital and business donations (*β* = 0.149, *β* = 0.141, *r*^2^ = 0.260) respectively, have effect on SMEs profit growth (Table 3).

**Table 2:** Standard multiple regression of financing strategies and technology-based SMEs business growth.

	**Profit growth**		**Sales growth**		**Employee growth**	
**Ind. variable**	**β**	**P-value**	**β**	**P-value**	**β**	**P-value**
Personal savings	−.076	.411	−.026	.786	−.052	.599
Bank loan	.109	.255	.037	.703	.048	.635
Venture capital	.149	.087*	.033	.713	.127	.165
Business Donations	.141	.071*	−.019	.815	.132	.110
*R*	0.260		.047		.202	
*R*^2^	.067		.002		.041	
Adj *R*^2^	.051		−.015		.024	
F-value	4.125 (4, 228); p = 0.003		.124 (4, 228); p = 0.974		2.421 (4, 228); p = 0.049	

* *p* ≤ 0.1

**Table 3:** Standard multiple regression of entrepreneurial competencies and financial strategies of technology-based SMEs.

	**Bank loan**		**Venture lapital**		**Business lonations**	
**Ind. variable**	**β**	**P-value**	**β**	**P-value**	**β**	**P-value**
Skills	.152	.011***	.186	.006***	.367	.000***
Attitude	.062	.307	.089	.197*	.184	.003***
Knowledge	.010	.871	−.019	.784	−.009	.885
R	0.230		.244		.502	
R^2^	.053		.059		.252	
Adj R^2^	.040		.047		.242	
F-value	4.264 (3, 229); *p* = 0.006		4.827 (3, 229); *p* = 0.003		25.696 (3, 229); *p* = 0.000	

** *p* ≤ 0.05,

* *p* ≤ 0.1,

*** *p* ≤ 0.01

Table 2 contains data on the influence of entrepreneurial capacities on accessibility to financing strategies by technology-based SMEs’ operators. From the data, skills significantly influences bank loan (*β* = 0.152), venture capital (*β* = 0.186) and business donations (*β* = 0.367). Attitude also significantly relates with venture capital (*β* = 0.089) and business donations (*β* = 0.184).

## 2 Experimental design, materials and methods

Survey method was adopted to gather data. 233 owners and financial managers of technology-based SMEs in Lagos State, Nigeria were included in the research. Technology-based SMEs are globally recognized to be significant to enhancing global economies, especially through their contribution to GDP, advancement of economic competitiveness and tax revenue generation [1], [2], [4], [3], [9]. However, in Nigeria, there is still dearth in literature, especially with data evidence about the financing strategies that are most significant to facilitating the activities of technology-based SMEs and the role of entrepreneurial competencies on their business growth [8], [10]. This research benefitted from the ideas of existing research studies. Questions that pertained to entrepreneurial competencies was developed based on [4], while items of SMEs’ growth was developed based on [5], [6]. Financing strategy questionnaire was developed based on [7]. The collated data were coded and entered in SPSS version 22. Data analysis was performed applying descriptive statistics and structural equation modelling (SEM). Ethical consideration in the research process was ensured because administering the questionnaires to respondents was based on their willingness to respond to the research instrument. Moreover, confidentiality and anonymity for participants in the study was assured.

## References

[1] S. Ojeka, F. Iyoha, A. Ibidunni, C. Ayo, D. Gberevbie, Role of e-Government in Nigeria’s Tax System: Strategy Perspective to Enhance Compliance. 16th European Conference on e-Government, July 16–17 Ljubljana, Slovania, 2016.

[2] Volery T., Mueller S., Von Siemens B. (2015). Entrepreneur ambidexterity: a study of entrepreneur behaviours and competencies in growth-oriented small and medium-sized enterprises. Int. Small Bus J..

[4] Wagener S., Gorgievski M., Rijsdijk S. (2010). Businessman or host? Individual differences between entrepreneurs and small business owners in the hospitality industry. Serv. Ind. J..

[3] Chapple K., Ann M., Gregory S., Dai Y., Pingkang Y. (2004). Gauging metropolitan “High Tech” and “I-Tech” activity. Econ. Dev. Q..

[5] Venkatraman N. (1989). Strategic orientation of business enterprises: the construct, dimensionality, and measurement. Manag. Sci..

[6] A.A. Babajide, F.O. Olokoyo, J.N. Taiwo, Evaluation of effects of banking consolidation on small business finance in Nigeria, in: Proceedings of the 23rd International Business Information Management Association Conference, 2016, pp. 12522–12540.

[7] Robb A.M., Coleman S. (2010). Financing strategies of new technology-based firms: a comparison of women-and men-owned firms. J. Technol. Manag. Innov..

[8] Osabuohien E.S., Efobi U.R. (2012). Technology diffusion and economic progress in Africa: challenges and opportunities. Disruptive Technol. Innov. Glob. Redesign: Emerg. Implic..

[9] Ibidunni A.S., Atolagbe T.M., Obi J., Olokundun M.A., Oke O.A., Amaihian A.B., Borishade T.T., Obaoye D. (2018). Moderating effect of entrepreneurial orientation on entrepreneurial competencies and performance of agrobased SMEs. Int. J. Entrep..

[10] Ogundana O.M., Okere W., Ayomoto O., Adesanmi D., Ibidunni S., Ogunleye O. (2016). ICT and accounting system of SMEs in Nigeria. Manag. Sci. Lett..

