# Supplementary material for: Data on the relationships between financing strategies, entrepreneurial competencies and business growth of technology-based SMEs in Nigeria
Source: Data Brief. 2018 Apr 3;18:988–91. doi: 10.1016/j.dib.2018.03.136 (PMC5996611; doi:10.1016/j.dib.2018.03.136)
Supplement: Supplementary file 1 — Supplementary material [file mmc1.doc]

Ibidunni, Ayodotun Stephen (PhD)

Covenant University,

Ota,

Ogun State,

Nigeria

21st March, 2018

The Editor,

Data In Brief,

Dear Sir,

**DECLARATION OF CONFLICT OF INTEREST**

I, Dr. Ibidunni, Ayodotun Stephen and my colleagues write to declare that there is no conflict of interest traceable to our data paper “***Data Evidence on the Relationships between Financing Strategies, Entrepreneurial Competencies and Business Growth of Technology-based SMEs in Nigeria.***”

Yours faithfully,


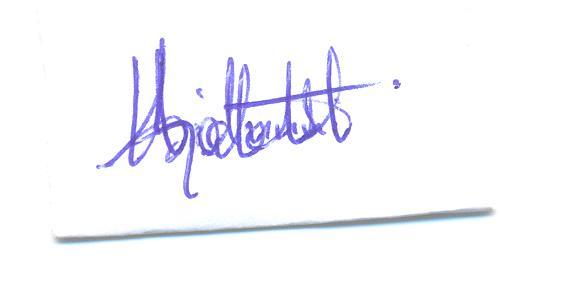


Ibidunni, Ayodotun Stephen (PhD) (Corresponding Author)

+234-803-489-3637
